# Impacts of long-term different fertilization regimes on microbial utilization of straw-derived carbon in greenhouse vegetable soils: insights from its ecophysiological roles and temperature responses

**DOI:** 10.3389/fpls.2024.1486817

**Published:** 2024-10-25

**Authors:** Long Ma, Ruonan Li, Haoan Luan, Jiwei Tang, Liying Wang, Tengfei Guo, Shaowen Huang

**Affiliations:** ^1^ State Key Laboratory of Efficient Utilization of Arid and Semi-arid Arable Land in Northern China/the Institute of Agricultural Resources and Regional Planning, Chinese Academy of Agricultural Sciences, Beijing, China; ^2^ Institute of Agricultural Resources and Environment, Hebei Academy of Agriculture and Forestry Sciences, Shijiazhuang, China; ^3^ College of Forestry, Hebei Agricultural University, Baoding, China; ^4^ Institution of Plant Nutrition and Environmental Resources, Henan Academy of Agricultural Sciences, Zhengzhou, China

**Keywords:** greenhouse vegetable soils, straw decomposition, long-term different fertilization treatments, incubation temperatures, DNA-SIP, high-throughput and metagenomic sequencing

## Abstract

As the largest organic carbon input in the agroecosystems, crop residues can increase soil carbon sequestration and crop production in greenhouse vegetable fields (GVFs). However, the soil microbiological mechanisms driving straw decomposition in GVFs under different incubation temperatures and fertilization treatments are not clear. Thus, soil samples were collected from a long-term field experiment included chemical fertilizer application alone (CF), 2/4 fertilizer N+2/4 organic fertilizer N (CM), 2/4 fertilizer N+1/4 organic fertilizer N+1/4 straw N (CMS), 2/4 fertilizer N+2/4 straw N (CS), and incubated with ^13^C-labeled straw at different temperatures (15, 25, and 35°C) for 60 days. Organic-amended treatments (CM, CMS, and CS), especially CMS treatment, increased soil bacterial *Alpha* diversity before and after straw addition. Straw decomposition process was dominated by soil *Proteobacteria*, *Actinobacteria*, and *Firmicutes* for each treatments. The effect of incubation temperature on soil microbial community composition was higher than that of fertilization treatments. Soil *Alphaproteobacteria* and *Actinomycetia* were the most predominant class involved in straw decomposition. *Gammaproteobacteria* (*Pseudomonas*, *Steroidobacter*, *Acidibacter*, and *Arenimonas*) were the unique and predominant class involved in straw decomposition at medium and high temperatures as well as in the straw-amended treatments. Organic-amended treatments, especially straw-amended treatments, increased the relative abundance of glycosyl transferases (GT) and auxiliary activities (AA). *Alphaproteobacteria*, *Actinomycetia*, and *Gammaproteobacteria* had higher relative contribution to carbohydrase genes. In summary, the long-term organic-amended treatments altered the structure of soil microbial communities and increased soil bacterial diversity, with the CMS having a greater potential to enhance resistance to external environmental changes. Soil *Alphaproteobacteria* and *Actinomycetia* were responsible for the dominance of straw decomposition, and *Gammaproteobacteria* may be responsible for the acceleration of straw decomposition. Fertilization treatments promote straw decomposition by increasing the abundance of indicator bacterial groups involved in straw decomposition, which is important for isolating key microbial species involved in straw decomposition under global warming.

## 1 Introduction

Recently, the issues of soil quality degradation in greenhouse vegetable fields (GVF) have been prominent, and the limiting factors are the imbalance of C vs. N inputs due to unreasonable fertilization treatments (i.e., excessive chemical N and organic manure (low C/N ratio inputs)) in the main GVF in China (Huang, 2019). The crop straw, characterized by low N contents and high C/N ratios, can coordinate the balance between soil nutrients (N) and energy (C), and maintain the sustainable production of GVF (Kamble et al., 2014; Huang, 2019). Currently, approximately 50 billion t of plant polymers (e.g., crop straw) per year are produced globally; meanwhile, the decomposition of these plant residues plays a crucial role in the C balance of terrestrial ecosystems (Wardle et al., 2004; Cornwell et al., 2008; Li et al., 2015; Kong et al., 2020). Several studies have pointed out that the chemical properties of plant residues, climate (e.g., temperature and precipitation), agricultural management measures (e.g., fertilization treatments and cultivation), and microbial traits are the main factors affecting the decomposition of plant residues (Bradford et al., 2017; Blesh and Ying, 2020; Yang et al., 2021). Among these factors, as the main biological driver for plant residue decomposition, dominant microorganisms play an important role in soil C cycling (Bradford et al., 2017; Xu et al., 2019; Blesh and Ying, 2020; Yang et al., 2021; Zheng et al., 2021). However, the inherent mechanism of how temperatures and fertilization treatments affect the microbial-driven degradation process of plant residues in the unique internal environment (e.g., high-temperature) of GVFs is still unclear.

The crop straw applied into soil undergoes microbial degradation, and its organic component begins to decompose and release nutrients (Tveit et al., 2013; Guo et al., 2022). During these decomposition processes, there are apparent changes in soil microorganisms at different stages, with significant differences in community structure composition. For instance, bacteria are more likely to utilize easily decomposed substrates (Marschner et al., 2011). The specific microorganisms were enriched in the soil after returning straw to the field, whether these microorganisms are assimilated to the carbon source of straw to promote their growth and reproduction or stimulated by organic materials to use the soil carbon source for reproduction and enrichment, it is not possible to investigate the microbial groups that are involved in the transformation of straw decomposition by using only the traditional methods (Murase et al., 2012). In addition, the critical microbiological processes that play a dominant role in straw decomposition are less investigated in GVPs. Thus, the study of microbial diversity and its ecological succession during straw decomposition under different fertilization treatments, and the in-depth exploration of the ecological functions and metabolic activities of key species will be a breakthrough in understanding the process of straw decomposition and its regulatory mechanisms, and will provide a scientific basis for the rational use of straw resources in the GVPs.

As a novel technology, DNA stable-isotope probing (DNA-SIP) can link environmental microorganisms with their specific functions at the microscopic level, which is beneficial for revealing the molecular mechanisms of vital physiological metabolic processes in a class of microorganisms (key species) with low abundance but performing critical functions in complex environments (Chen and Murrell, 2010; Banerjee et al., 2018; Guo et al., 2022);. For instance, based on the DNA-SIP technology, Yu et al. (2020) pointed out that actinomycetes own multiple genes involved in plant residue C degradation, e.g., endoglucanase, β-Glucosidase, α-Glucosidase, and α-Mannosidase; meanwhile, low abundance microbes may play a vital role in augmenting functional redundancy and enhancing the ability of microbes to resist environmental disturbance. Zhang et al. (2022) applied DNA-SIP technology to identify the soil microbial population involved in straw decomposition and found that the bacterial groups that assimilated and utilized straw C sources were mainly focused on *Actinobacteria*, *Firmicutes*, and *Proteobacteria*. Recently, several studies indicated that fertilization adapts to environmental changes by altering the microbial community structure’s composition and metabolic potential, thereby forming unique soil microbial community characteristics (Walker et al., 2018). Soil microorganisms responded more rapidly to straw application in the organic-amended treatments than in the chemical fertilizer application alone, and the composition of dominant soil microorganisms that assimilated the straw carbon source was different due to differences in cropping systems and climate (Zhan et al., 2018; Fan et al., 2019; Guo et al., 2022). Nevertheless, current studies have mostly focused on the differences in the microbial community composition during plant residue decomposition (Zhang et al., 2022; Guo et al., 2022); however, there is little information about the potential microbial physiological mechanisms underlying the above-mentioned microbial changes.

Thus, our study hypothesized that i) there may be some microbes in soils that promote straw decomposition during long-term straw application periods or at medium to high temperatures, and ii) the ecological functionality of dominant bacteria with higher relative abundance is stronger under different incubation temperatures and fertilization treatments. To verify these hypotheses, we collected soil samples from a long-term experiment with different fertilization treatments in GVFs. After adding ^13^C labeled straw, incubation experiments were conducted at different temperatures (15, 25, and 35°C) for 60 days. Besides, DNA-SIP technology combined with amplicon sequencing and metagenomic sequencing was used to analyze the differences in soil bacterial community structure and functionality during straw decomposition under different incubation temperatures and fertilization treatments.

## 2 Materials and methods

### 2.1 Experimental site description and soil sampling

The study was established in a greenhouse vegetable field at the Dahe experimental station, in Hebei Province, China (38°08′N, 114°23′E) in 2009, with winter-spring cucumber (*Cucumis sativus Lcv. Bomei No. 11*) and autumn-winter tomato (*Lycopersicum esculentum Mill.* cv. *Jinpeng No. 11*) rotation system. The experiment included four treatments: (i) chemical fertilizer application alone (CF), (ii) 2/4 fertilizer N+2/4 organic fertilizer N (CM), (iii) 2/4 fertilizer N+1/4 organic fertilizer N+1/4 straw N (CMS), and (iv) 2/4 fertilizer N+2/4 straw N (CS). The total amounts of nutrient (N, P_2_O5, and K_2_O) applied to each treatment was equal. For more information on field location experiments, see Rong et al. (2018). The specific N and C inputs in each fertilization treatment are shown in **Supplementary Table S1**.

Soil samples (0–20 cm) were randomly collected from ten positions in each plot in June 2021 (the 24^th^ cultivation season). These samples were placed on ice and transferred to the laboratory. Soil samples were sieved through a 2 mm mesh after the removal of stones and plant residues. A part of soil samples was used for incubation experiments. The other parts were used to determine the bioinformatic analyses and basic physicochemical parameters.

### 2.2 Laboratory incubation

Two factors were designed for the incubation experiments, i.e. incubation temperature (15, 25, and 35°C) and fertilization treatment (CF, CM, CMS, and CS). The soil samples (equivalent to 20 g dry soil) and 0.1 g ^13^C-labeled maize straw (94.9 atom% ^13^C) were thoroughly mixed and transferred to 100 mL unsealed glass bottles. Simultaneously, a soil sample (CK) with ^12^C-labeled maize straw was also prepared. The treatments were set up in 36 replicates (3 incubation temperatures × 3 plot samples × 4 incubation periods). Dark aerobic incubation experiments were carried out in artificial climate chambers at 15, 25, and 35°C with soil moisture content maintained at 75% of field capacity for 60 days. Our previous research found that the 7 days of straw decomposition is a period of rapid decomposition, and straw decomposition is basically over after 30 days (Ma et al., 2024). Therefore, we analyzed soil samples on the 7^th^ and 30^th^ day of the incubation period.

### 2.3 DNA extraction, gradient fractionation and quantitative PCR analysis

Soil DNA was isolated from 0.50 g of fresh soil using FastDNA SPIN Kit DNA on a Fast Prep-24 Homogenisation System (MP Biomedicals, Irvine, CA, United States) as described by the manufacturer. DNA concentration was determined by Nanodrop spectrophotometer (Nanodrop, Peq Lab, Germany).

Soil DNA extracted from the addition of ^3^C-straw and ^1 12^C-straw treatments on the 7^th^ and 30^th^ day of incubation was stratified by ultra-highspeed centrifugation (Guo et al., 2022). For each sample, we mixed soil DNA (3 μg), CsCl (1.85 g ml^-1^), and a gradient buffer (1 mM EDTA, 0.1 M KCl, 0.1 M Tris-HCl, pH = 8.0) to achieve a final mixture density of 1.725 g ml^-1^. The solution was centrifuged for 44 h (190,000 × g) at 20°C by a Vti65.2 vertical rotor (Beckman Coulter Inc., Palo Alto, CA, United States) and Quick-Seal polyallomer ultracentrifugation tubes (5.1 ml). After centrifugation, the solution in the centrifuge tube was divided into 15 equal portions (380 μl each) using a syringe pump. The density of each buoyant was determined by a digital hand-held refractometer. We purified the fractionated DNA samples and then re-eluted them with 30 μl of sterilized ultrapure water for subsequent analyses.

The 16S rRNA genes of each soil microcosm system were analyzed by qPCR in triplicate using the iCycler system (Bio-Rad). The reaction system (20 μl) included 2.0 μl of DNA, 4 μM of each primer, 10 μl qPCR Master Mix (Vazyme Biotech Co., Ltd.). Primers 515F (5’-GTGCCAGCMGCCGCGGTAA-3’) and 806R (5’-GGACTACHVGGGTWTCTAAT-3’) (Zhang et al., 2022).

### 2.4 Amplicon high-throughput sequencing analysis

To reveal taxonomic characteristics (ecological attributes), we identified bacterial communities involved in straw decomposition on Illumina 16S rRNA gene sequencing by Novogene Co., Tianjin, China. A total of 228 (72 from the ^13^C treatments after gradient fractionation, 72 from the ^12^C treatment after gradient fractionation, 72 from the ^13^C treatments before gradient fractionation, and 12 from before the start of the incubation experiment) DNA composite samples were selected for amplicon sequencing by targeting the V4 region of the 16S rRNA gene with the primer sets 515F (5’-GTGCCAGCMGCCGCGGTAA-3’) and 806R (5’-GGACTACHVGGGTWTCTAAT-3’). Sequences analysis was conducted using Uparse software (Edgar, 2013). Sequences with ≥97% similarity were grouped into the same OTU and representative sequences from each OTU were screened for further annotation. For each representative sequence, the Silva Database (https://www.arb-silva.de/)was used based on Mothur algorithm to annotate taxonomic information (Quast et al., 2012). All raw reads were archived in the NCBI Sequence Read Archive database (accession number: PRJNA1089403).

### 2.5 Shotgun metagenomic sequencing analysis

To reveal bacterial functional profiling (physiological attributes), DNA-SIP-based shotgun metagenomic sequencing was conducted on Illumina NovaSeq/Hiseq Xten at Majorbio Bio-Pharm Technology Co., Ltd. (Shanghai, China). A total of 36 samples were selected from the ^13^C treatments DNA composite samples after gradient fractionation for library construction and shotgun metagenomic sequencing. Extracted DNA was fragmented at an average of 400 bp (Genetics Co. Ltd., China) to construct paired-end libraries using Covaris M220. Extracted DNA was fragmented at an averagely of about 400 bp through Covaris M220 (Gene Co., Ltd., China) to construct paired-end libraries. Data were analyzed on the Majorbio Cloud Platform (www.majorbio.com) free online platform. Representative sequences from the non-redundant gene catalogue were aligned to the NR database using Diamond for taxonomic annotation (Buchfink et al., 2015). Carbohydrate-active enzymes (CAZy) annotation was conducted using hmmscan (http://hmmer.janelia.org/search/hmmscan) against the CAZy database. About 10 Gbp of Illumina data were obtained for each sample. All raw reads were archived in the NCBI Sequence Read Archive database (accession number: PRJNA1089381).

### 2.6 Soil physicochemical analysis

Soil nitrate-N (NO_3_^−^-N) was extracted by 2 M KCl and measured using a flow injection autoanalyzer (Smartchem 200, Alliance, Paris, France) (Norman et al., 1985). Soil total nitrogen (TN) and organic carbon (SOC) were determined through an elemental analyzer (Elementar Analysensy steme GmbH, Hanau, Germany). Soil pH was determined using a pH meter (Mettler Toledo, Switzerland) with a water/soil ratio of 2.5:1. Soil available phosphorus (P) and available potassium (K) were measured by the Olsen method and the flame photometry method, respectively (Olsen et al., 1954; Helmke and Sparks, 1996).

### 2.7 Statistical analysis

The Kruskal–Wallis H test was performed with STAMP statistics (https://beikolab.cs.dal.ca/software/STAMP) to compare the abundance of OTU -indicators in the ^13^C-treatment samples with those in the ^12^C-treatment samples.

One-way analysis of variance (ANOVA) and tests of multiple comparisons across treatments (Duncan’s *post hoc* test, *P <*0.05) were performed on the measurements under different treatments using IBM SPSS statistical software (SPSS, Inc., Chicago, IL, USA). Principal coordinate analysis (PCoA) was performed based on the Bray–Curtis dissimilarity of bacterial communities using the “vegan” packages of R. Linear discriminant analysis (LDA) effect size analyses were performed using the LEfSe tool (https://bioincloud.tech/standalone-task-ui/lefse). Correlation analyses between species abundance and functional abundance based on the relative species and functional abundance of samples to identify the functional contribution of specific species were analyzed on the platform (www.i-sanger.com) provided by Majorbio Co., Ltd. (Shanghai, China).

## 3 Results

### 3.1 Soil microbial community structure under long-term different fertilization treatments

**Figure 1:**
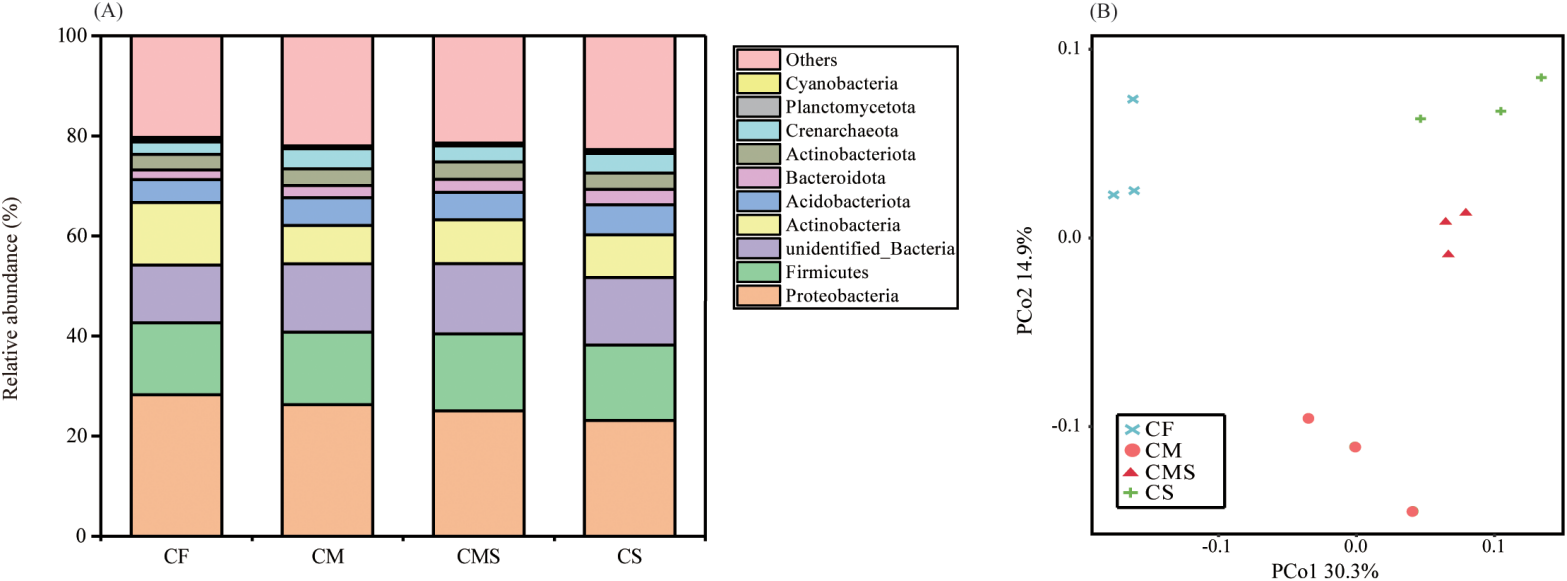
The relative abundance at phylum level for bacterial **(A)** under different fertilization treatments. PCoA analysis of bacterial communities **(B)** under different fertilization treatments. .

As shown in **Figure 1A**, the dominant bacterial phylum under different fertilization treatments was *Proteobacteria*, which comprised 25.7% of the total sequences on average, followed by *Firmicutes*, *Actinobacteria*, and *Acidobacteria*, which represented 14.8%, 9.7%, and 5.4% of the sequences, respectively. Compared to the chemical fertilizer application alone (CF), organic-amended treatments (CM, CMS, and CS) increased the relative abundance of Firmicutes, Bacteroidetes, Acidobacteria, Gemmatimonadetes, and Crenarchaeota by an average of 4.1%, 43.9%, 23.2%, 9.1%, and 48.6%, respectively. In contrast, the relative abundance of *Proteobacteria* and *Actinobacteria* were lower in organic-amended treatments than in CF treatment.

Principal coordinates analysis (PCoA; **Figure 1B**) of soil bacteria at the OTU level for the 12 samples shows that the PCo1 and PCo2 explained 30.3% and 14.9% of the total variance in bacterial community profiles, respectively. Meanwhile, the soil samples were distinctly separated into three groups, i) CF, ii) CM, and iii) CMS and CS. Compared to CF treatment, organic-amended treatments, especially straw-amended treatments (CMS and CS), exhibited higher bacterial *Alpha* diversity (as indicated by the higher values of Shannon, Chao1, and Ace; **Table 1**).

**Table 1:** Soil bacterial *Alpha* diversity index under different fertilization treatments.

Fertilization treatments	Shannon	Chao1	Ace
CF	9.8 ± 0.1b	4438.4 ± 145.5b	4519.8 ± 103.0c
CM	10.0 ± 0.0ab	4552.9 ± 16.5ab	4591.4 ± 26.8bc
CMS	10.1 ± 0.0a	4738.4 ± 46.5a	4792.9 ± 61.3a
CS	10.1 ± 0.1a	4666.0 ± 58.9a	4702.6 ± 55.6ab

Different lowercase letters indicate significant differences at P < 0.05 for the different fertilization treatments.

### 3.2 Soil microbial community structure during straw decomposition

**Figure 2:**
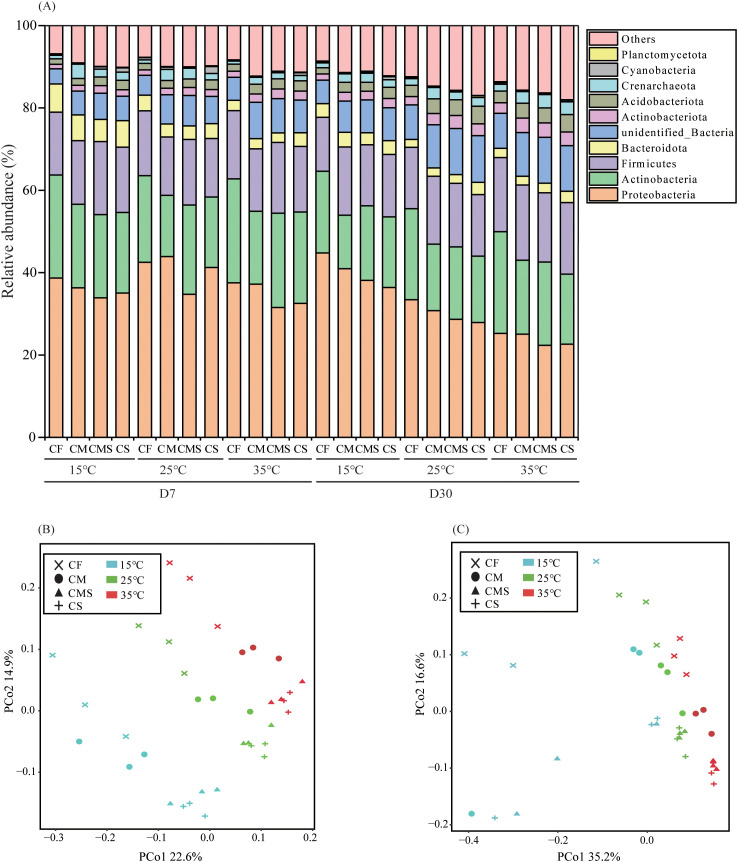
The relative abundance at bacterial phylum level **(A)** during straw decomposition processes under different fertilization treatments and incubation temperatures.PCoA analysis of bacterial communities on the 7^th^ **(B)** and 30^th^ **(C)** days of straw decomposition under different fertilization treatments and incubation temperatures. .

As shown in **Figure 2A**, there are five dominant bacterial phyla (relative abundance > 1.0%) under different incubation temperatures and fertilization treatments. *Proteobacteria* had the highest relative abundance (22.6%-44.8%), followed by *Actinobacteria* (13.0%-25.2%), *Firmicutes* (13.1%-18.3%), *Bacteroidetes* (1.9%-6.87%), and *Acidobacteria* (1.3%-4.3%).

Principal coordinates analysis (PCoA) as conducted on soil bacteria from 36 samples on the 7^th^ and 30^th^ days of straw decomposition at the OTU level (**Figures 2B, C**). The results showed that PCo1 and PCo2 explained 22.6% and 14.9% of the total variance in bacterial community profiles on the 7^th^ day, respectively, and 35.2% and 16.6% of the total variance on the 30^th^ day, respectively. On the 7^th^ day of incubation, the bacterial community structure at different temperatures can be divided into three groups: i) 15°C, ii) 25°C, and iii) 35°C; meanwhile, the bacterial community structure under different fertilization treatments can also be divided into three groups: i) CF, ii) CM, and iii) CMS and CS. On the 30^th^ day of incubation, the bacterial community structure at different temperatures can be divided into two groups: i) 15°C and ii) 25°C and 35°C; additionally, the fertilization treatments have no significant impact on the composition of bacterial community structure.

The Chao1 and Ace in the soil bacterial *Alpha* diversity were significantly higher at 15°C than at 25°C and 35°C on the 7^th^ day, and incubation temperature did not significantly affect soil bacterial *Alpha* diversity on the 30^th^ day. As compared to CF treatment, organic-amended treatments, especially straw-amended treatments, exhibited higher bacterial *Alpha* diversity (as indicated by the higher values of Shannon, Chao1, and Ace; **Table 2**).

**Table 2:** Soil bacterial *Alpha* diversity index during straw decomposition processes under different fertilization treatments and incubation temperatures.

Incubation days			15°C	25°C	35°C	Mean
7^th^ day	Shannon	CF	7.8 ± 0.3Ac	8.3 ± 0.2Ab	8.1 ± 0.2Ab	8.1 ± 0.3b
		CM	8.5 ± 0.3Bb	9.1 ± 0.3Aa	9.2 ± 0.3Aa	8.9 ± 0.4a
		CMS	9.0 ± 0.2Aav	9.0 ± 0.0Aa	9.0 ± 0.0Aa	9.0 ± 0.1a
		CS	9.1 ± 0.0Aa	9.1 ± 0.2Aa	9.1 ± 0.1Aa	9.1 ± 0.1a
		Mean	8.6 ± 0.6A	8.9 ± 0.4A	8.8 ± 0.5A	8.8 ± 0.5
	Chao1	CF	3556.3 ± 118.0Ab	3429.9 ± 112.3Ab	3358.7 ± 168.6Ab	3448.3 ± 158.1c
		CM	3917.8 ± 76.5Aab	3495.3 ± 131.8Bb	3625.0 ± 83.7Bab	3679.4 ± 203.2bc
		CMS	3998.7 ± 452.0Aab	3581.9 ± 66.8Aab	3677.3 ± 68.5Aa	3752.6 ± 320.9ab
		CS	4318.9 ± 155.4Aa	3785.0 ± 12.6Ba7	3884.1 ± 121.2Ba	3996.0 ± 258.4a
		Mean	3947.9 ± 368.3A	3573.0 ± 162.9B	3636.3 ± 220.9B	3719.1 ± 311.7
	Ace	CF	3774.4 ± 217.3Ab	3588.6 ± 36.1Ac	3538.7 ± 189.8Ab	3633.9 ± 196.1c
		CM	4060.9 ± 98.2Aab	3640.2 ± 111.0Bbc	3705.6 ± 132.3Bab	3802.2 ± 217.6bc
		CMS	4142.4 ± 398.3Aab	3826.0 ± 81.2Aab	3909.7 ± 73.6Aa	3959.3 ± 273.5ab
		CS	4516.7 ± 47.9Aa	3984.4 ± 92.6Ba	4051.4 ± 169.9Ba	4184.2 ± 263.2a
		Mean	4123.6 ± 353.1A	3759.8 ± 178.4B	3801.3 ± 245.1B	3894.9 ± 314.0
30^th^ day	Shannon	CF	8.6 ± 0.2Ab	8.8 ± 0.1Ab	8.7 ± 0.2Ab	8.7 ± 0.2c
		CM	8.9 ± 0.2Aab	9.2 ± 0.2Aa	9.1 ± 0.1Aa	9.1 ± 0.2b
		CMS	9.2 ± 0.1Ba	9.5 ± 0.0Aa	9.2 ± 0.1Ba	9.3 ± 0.2a
		CS	9.3 ± 0.2Aa	9.6 ± 0.2Aa	9.4 ± 0.1Aa	9.4 ± 0.1a
		Mean	9.0 ± 0.4A	9.3 ± 0.3A	9.1 ± 0.3A	9.1 ± 0.1
	Chao1	CF	3402.5 ± 174.7Ab	3627.9 ± 201.8Aa	3672.3 ± 99.0Ac	3567.6 ± 202.4c
		CM	3828.9 ± 121.3Aa	3779.8 ± 215.1Aa	3719.8 ± 31.1Abc	3776.2 ± 150.5b
		CMS	3927.9 ± 64.8Aa	3980.0 ± 97.2Aa	3842.9 ± 40.8Aab	3850.3 ± 201.6ab
		CS	3965.8 ± 92.7Aa	3982.5 ± 258.3Aa	3966.9 ± 63.1Aa	3971.7 ± 162.8a
		Mean	3781.3 ± 176.3A	3842.6 ± 250.8A	3800.5 ± 131.2A	3808.4 ± 233.0
	Ace	CF	3481.3 ± 190.7Ab	3679.2 ± 211.4Aa	3718.7 ± 106.6Ac	3626.4 ± 203.9c
		CM	3894.5 ± 126.9Aa	3826.9 ± 199.5Aa	3788.9 ± 30.2Abc	3836.7 ± 144.4b
		CMS	3979.0 ± 73.4Aa	4040.3 ± 107.0Aa	3902.5 ± 54.1Aab	3918.4 ± 191.2ab
		CS	4038.5 ± 91.4Aa	4026.6 ± 270.1Aa	4019.6 ± 67.4Aa	4028.2 ± 169.3a
		Mean	3848.3 ± 272.5A	3893.2 ± 254.2A	3857.4 ± 134.2A	3866.3 ± 231.5

Different lowercase letters indicate significant differences at P < 0.05 for the different fertilization treatments under the same temperature condition. Different uppercase letters indicate significant differences at P < 0.05 for the different temperatures under the same fertilization treatment.

### 3.3 Soil microbial community structure utilizing straw carbon sources

On the 7^th^ day of incubation, 67-538 bacterial OTUs exist in the ^13^C-heavy DNA under different incubation temperatures and fertilization treatments. These bacterial OTUs were dominated by *Proteobacteria* (30.1%-64.4%) and *Actinobacteria* (16.2%-67.7%), followed by *Bacteroidetes* (0.1%-10.2%), *Firmicutes* (0.0%~14.3%), *Chlorobacteria* (0.1%~2.3%), *Verrucomycota* (0.0%~1.5%), and *Acidobacteria* (0.0%~0.9%). On the 30^th^ day of incubation, there are 106-470 bacterial OTUs in the ^13^C-heavy DNA under different incubation temperatures and fertilization treatments. These bacterial OTUs were dominated by *Proteobacteria* (25.4%-63.7%) and *Actinobacteria* (2.5%-64.9%), followed by *Firmicutes* (0.1%-20.6%), *Bacteroidetes* (0.1%-12.9%), *Chloroflexi* (0.0%-2.3%), *Verrucomycota* (0.0%-0.7%), and *Acidobacteria* (0.0%-1.4%).

**Figure 3:**
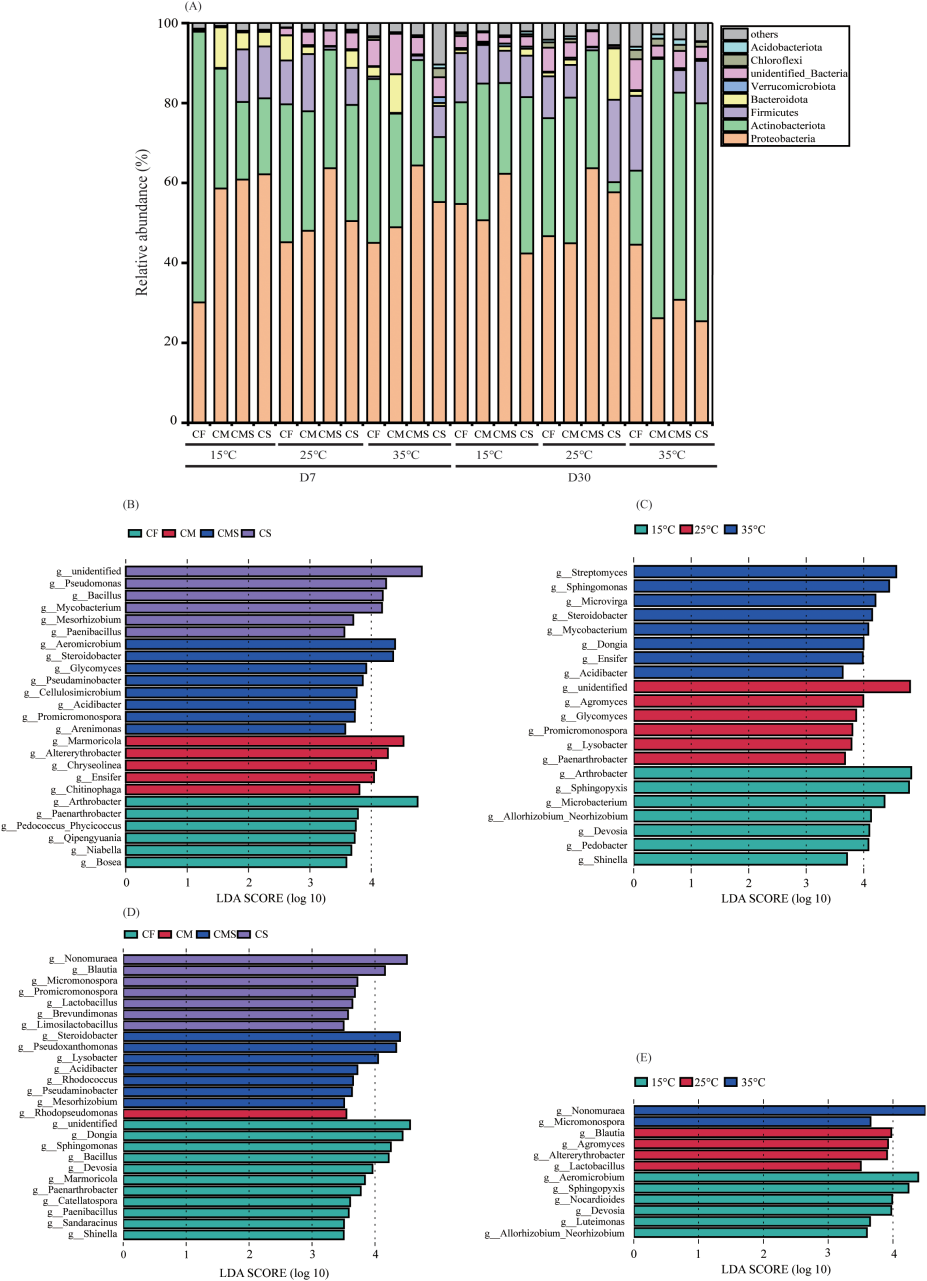
The relative abundance at bacterial phylum level of ^13^C-labeled OTUs **(A)** during straw decomposition processes under different fertilization treatments and incubation temperatures. Indicator bacterial groups at genes level with higher LDA values than 3.5 on the 7^th^ day **(B, C)** and 30^th^ day of straw decomposition **(D, E)**.

As shown in **Figure 3A**, the relative abundance of soil *Verrucomicrobia*, *Chloroflexi*, and *Acidobacteria* involved in straw decomposition tended to increase with increasing temperature on the 7^th^ day, and the relative abundance of *Actinomycetes* showed a decreasing trend. The organic-amended treatments, especially straw-amended treatments, increased the relative abundance of soil *Proteobacteria*, *Firmicutes*, and *Acidobacteria*, and decreased the relative abundance of *Actinobacteria* compared to CF treatment. The relative abundance of soil *Chlorobacteria* and *Acidobacteria* involved in straw decomposition tended to increase with increasing temperature on the 30^th^ day, whereas the relative abundance of *Verrucomicrobia* and *Firmicutes* tended to decrease. Compared to the CF treatment, organic-amended treatments, especially straw-amended treatments, increased the relative abundance of soil *Actinobacteria*, and decreased the relative abundance of *Chloroflexi*.

We identified specific phylotypes responding to different incubation temperatures and fertilization treatments at different sampling times by using LEfSe (**Figures 3B–E**). On the 7^th^ day of incubation, *Alphaproteobacteria* and *Actinomycetia* were the common and most predominant class involved in straw decomposition under different incubation temperatures and fertilization treatments. *Gammaproteobacteria* (*Pseudomonas*, *Steroidobacter*, *Acidibacter*, and *Arenimonas*) were the unique and predominant class involved in straw decomposition at medium and high temperatures (25°C and 35°C) as well as in the straw-amended treatments (CMS and CS). On the 30^th^ day of incubation, *Actinomycetia* was the common and most predominant class involved in straw decomposition under different incubation temperatures, and *Alphaproteobacteria* was the common and most predominant class involved in straw decomposition under different fertilization treatments.

**Figure 4:**
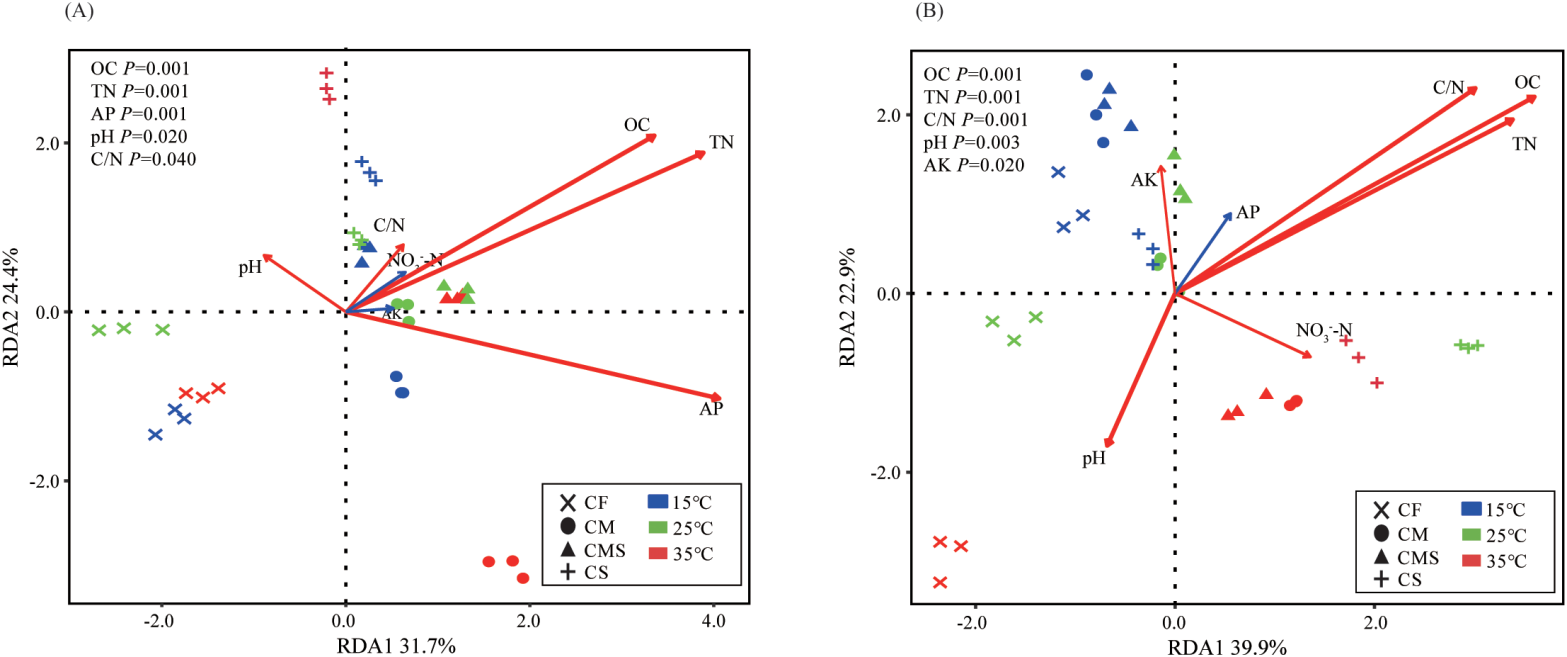
Redundancy analysis (RDA) of soil bacterial genus level of ^13^C-labeled OTUs by soil physicochemical properties on the 7^th^ day **(A)** and 30^th^ day **(B)** of straw decomposition under different fertilization treatments and incubation temperatures. OC, organic carbon; TN, total nitrogen; AP, Available P; AK, Available K.

According to the redundancy analysis (RDA) of the bacterial community structure constrained by soil physico-chemical properties, OC (*P*=0.001), TN (*P*=0.001), and AP (*P*=0.001) are the main environmental factors affecting the bacterial community structure on the 7^th^ day of incubation (**Figure 4A**). Meanwhile, OC (*P*=0.001), TN (*P*=0.001), and C/N (*P*=0.001) are the main environmental factors affecting the bacterial community structure on the 30^th^ day of incubation (**Figure 4B**). In sum, the bacterial community structure involved in straw decomposition has a stronger response to OC and TN during each incubation period.

### 3.4 Soil carbohydrase genes utilizing straw carbon sources

**Figure 5:**
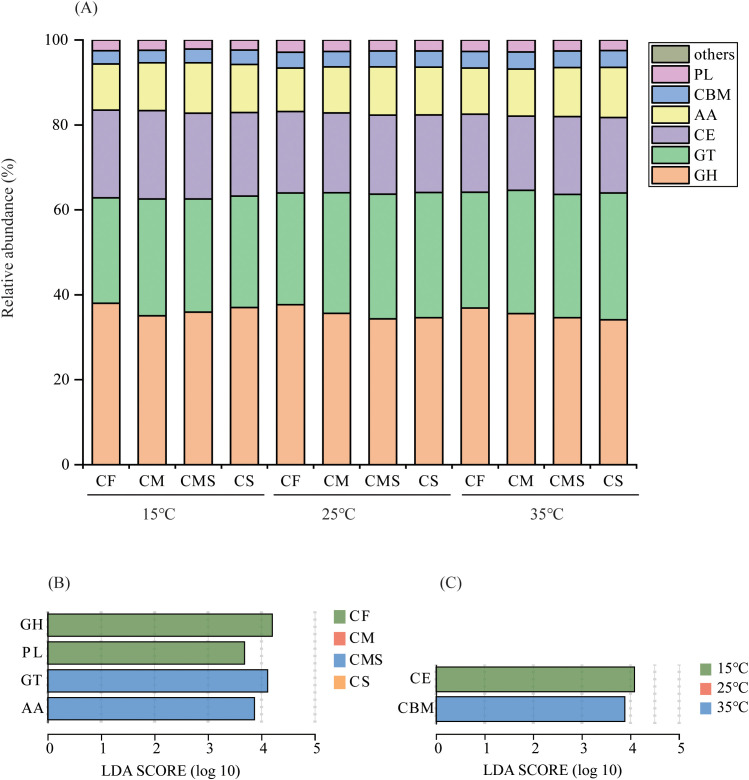
The relative abundance at class level of ^13^C-labeled CAZymes **(A)** on the 7^th^ day of straw decomposition under different fertilization treatments and incubation temperatures. Indicator CAZymes groups at class level with higher LDA values than 3.5 on the 7^th^ day **(B, C)** of straw decomposition. GH, Glycoside Hydrolases; GT, Glycosyl Transferases; CE, Carbohydrate Esterases; AA, Auxiliary Activities; CBM, Carbohydrate-Binding Modules; PL, Polysaccharide Lyases.

As shown in **Figure 5A**, the relative abundance of glycosyl transferases (GT), carbohydrate-binding modules (CBM), and polysaccharide lyases (PL) enzyme genes involved in straw decomposition tended to increase with increasing temperature on the 7^th^ day of incubation, whereas the relative abundance of glycoside hydrolases (GH) and carbohydrate esterases (CE) enzyme genes tended to decrease. The organic-amended treatments, especially straw-amended treatments, increased the relative abundance of GT and auxiliary activities (AA) enzyme genes and decreased the relative abundance of GH, CE, and PL enzyme genes. Significant markers of CAZymes involved in straw decomposition were GH and PL in CF treatment, and GT and AA in CMS treatment (**Figure 5B**). Significant markers of CAZymes involved in straw decomposition were CE at 15°C, and CBM at 35°C (**Figure 5C**).

**Figure 6:**
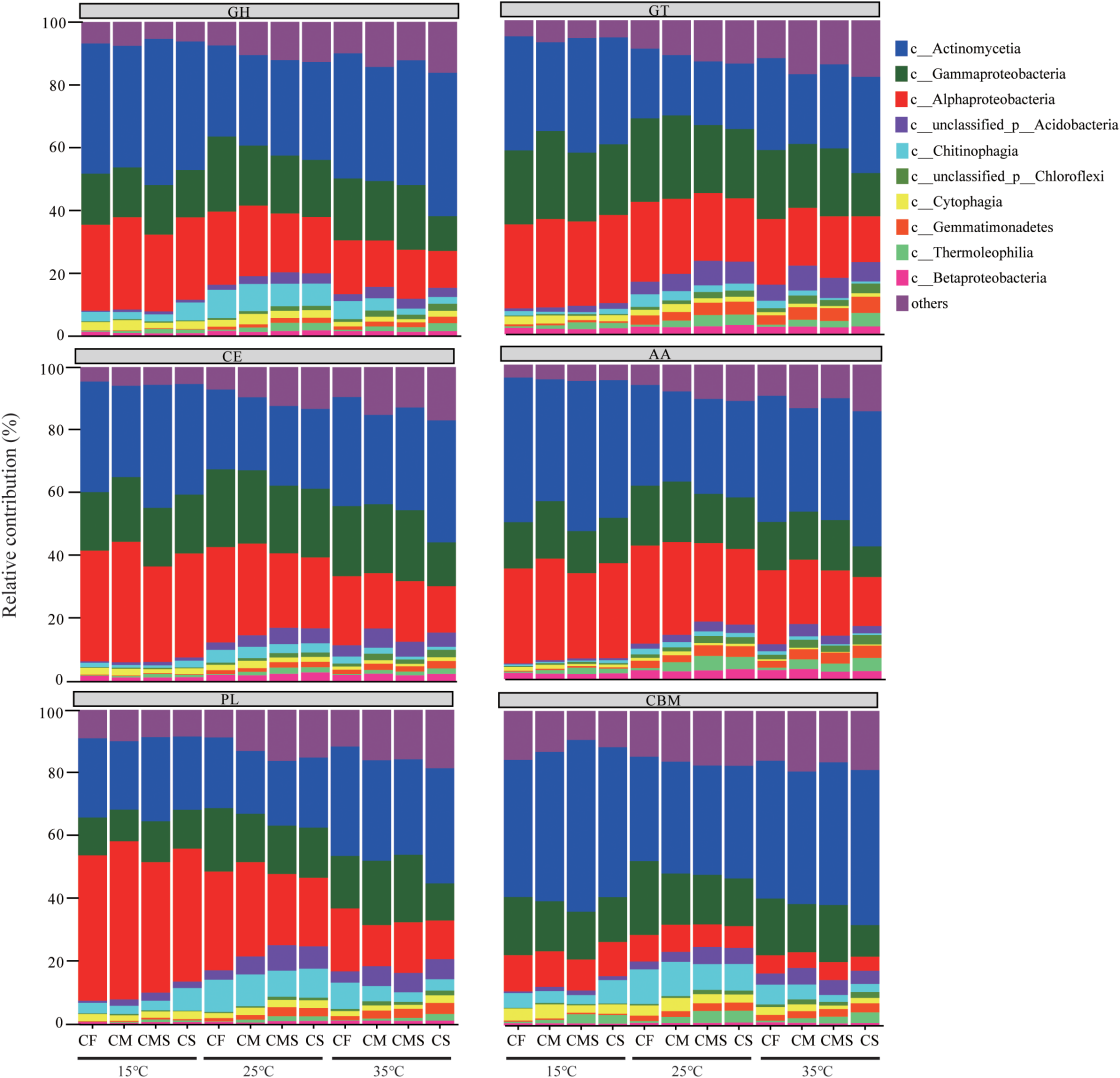
Phylogenetic distributions of CAZymes in the dominant bacterial class possessing CAZymes encoding-genes. GH, Glycoside Hydrolases; GT, Glycosyl Transferases; CE, Carbohydrate Esterases; AA, Auxiliary Activities; CBM, Carbohydrate-Binding Modules; PL, Polysaccharide Lyases.

As shown in **Figure 6**, the dominant bacteria with a higher proportion have a higher contribution to CAZymes under different incubation temperatures and fertilization treatments. The sum of the relative contributions of dominant bacteria (*Actinobacteria*, *Alphaproteobacteria*, and *Gammaproteobacteria*) to GH, GT, CE, AA, PL, and CBM is averagely 75.9%, 72.6%, 78.2%, 79.1%, 70.7%, and 67.1%, respectively.

## 4 Discussion

### 4.1 Effects of long-term organic-amended treatments on soil microbial communities

Fertilization can affect soil microbial proliferation and functional metabolism by altering soil nutrient conditions and physical properties (Mi et al., 2016; Wei et al., 2017). Researches-based on grain crops (corn, wheat, and rice) and vegetables (open fields and facilities) have shown that long-term organic fertilization (i.e., crop straw and manure) were beneficial for increasing microbial diversity (Ai et al., 2018; Luan et al., 2020; Jin et al., 2022; Wang et al., 2023). Our results also indicated that organic-amended treatments increase bacterial diversity as indicated by Shannon, Chao1, and Ace. Moreover, we found that the CMS treatment (i.e., high C diversity), rather than CS treatment (i.e., high C quantity), were more conducive to enriching bacterial diversity. The likely reason for these findings was that organic fertilizer or straw is rich in carbon and nitrogen resources, which not only provides “food” for microorganisms after being put into the soil, but also improves the soil structure, thus providing a more suitable “micro-environment” for microbial growth and reproduction (Mi et al., 2016; Wei et al., 2017). Organic fertilizer or straw application shifts the structural composition of soil microbial communities towards fast-growing communities (symbiotic nutrient groups), increases the diversity and sustainability of soil microbial communities, and facilitates the formation of soil microbial taxa associated with healthy crop growth (Reilly et al., 2013; Gonthier et al., 2014; Lupatini et al., 2017). There is an equilibrium point between soil organic carbon input and mineralization, when the equilibrium point is reached, the soil organic carbon will not increase and reach the threshold, the soil microbial growth and reproduction also exists in this law (Zhang et al., 2020). Therefore, in our study, the diversity of the soil microbial communities did not increase with an increase in carbon inputs. Combining manure with straw makes it easier to form a nutrient-rich environment, which can provide more effective nutrients and energy substances for the more active symbiotic flora. In our study, the soil microbial *Alpha* diversity index of the CMS treatment was the highest and had the strongest potential to withstand external environmental changes (Delgado-Baquerizo et al., 2016; Bei et al., 2018).

### 4.2 Effect of fertilization treatments and incubation temperatures on microbial communities utilizing straw carbon sources

Recently, several studies revealed that adding straw reduced the microbial diversity (Sanaullah et al., 2016; Zhao et al., 2019; Ye et al., 2022). This finding was confirmed by our study (**Tables 1**, **2**) and indicated that symbiotic populations stimulate a subset of the entire community, whereas other subsets may not respond to straw, leading to a decrease in microbial community diversity (Tardy et al., 2015; Sanaullah et al., 2016). Meanwhile, we found that the response of soil microbial diversity to different fertilization treatments was consistent with that before straw addition, i.e., soil microbial diversity was higher in the organic-amended treatments than in the chemical fertilizer application alone. Previous research on the microbial diversity during straw decomposition at different temperatures has shown that the diversities of the bacterial community have no significant response to temperature during straw decomposition (Wahdan et al., 2023). Another study reveals that the microbial diversity index is higher under low- and medium-temperature treatments than under high-temperature treatments in the early stage of straw decomposition; meanwhile, the bacterial community diversity index was the highest under medium-temperature treatment in the later stage of straw decomposition (Zhou et al., 2016). Our research results indicate that in the early stage of straw decomposition, the diversity of soil bacterial communities decreases with increasing temperature. In the later stage of straw decomposition, there was no significant difference in the diversity index of soil bacterial communities at different incubation temperatures, with the highest diversity index at 25°C. In the early stage of straw decomposition, the soil bacterial community has a high reactivity to climate change (temperature), which explains our results (Glassman et al., 2018).

Long-term organic application can improve soil microbial community structure, enhance microbial activity, and thereby affect straw decomposition (Fang et al., 2018). The results of previous studies showed that the soil microorganisms of straw decomposition were dominated by soil *Proteobacteria*, *Actinobacteria*, *Firmicutes*, and *Bacteroidetes* in the soil of grain field crops (corn, wheat, and rice) through DNA-SIP (Fan et al., 2019; Kong et al., 2020; Guo et al., 2022; Zhang et al., 2022). Meanwhile, there are variable effects of organic-amended treatments on different dominant phyla in straw decomposition. Guo et al. (2022) indicated that soil *Bacteroidetes* (*Flavobacterium*) was an indicator bacterial group for the straw-amended treatments, whereas *Chloroflexi (Herpetosiphon*) and *Gammaproteobacterial* (*Psudoxanthomonas*) were indicator bacterial groups for the chemical fertilizer application alone. Partial results of our study support the above research that microorganisms dominating straw decomposition mainly contain *Proteobacteria*, *Actinobacteria*, *Firmicutes*, and *Bacteroidetes.* Meanwhile, we found that *Alphaproteobacteria* and *Actinomycetia* were the common and most predominant class involved in straw decomposition under different fertilization treatments during the period of rapid straw decomposition, and *Gammaproteobacteria* (*Pseudomonas*, *Steroidobacter*, *Acidibacter*, and *Arenimonas*) were the unique and predominant class involved in straw decomposition in the straw-amended treatments (CMS and CS). When resource conditions are favorable, eutrophic groups (*Proteobacteria*, *Actinobacteria*, *Firmicutes*, and *Bacteroidetes*) have higher nutrient requirements, preferentially consume unstable soil organic carbon pools, show higher growth rates, and have an increased proportion in the soil (Fazi et al., 2005). Our result confirmed the first hypothesis. Some soil bacterial phyla show a tendency to be symbiotic and have high abundance in soils with high organic carbon, *Proteobacteria* and *Bacteroidetes* usually show co-nutritional properties in combination with organic carbon-rich substrates; *Actinobacteria* possess several genes related to carbon degradation in plant residues, namely endoglucanases, β-glucosidases, α-glucosidases, and α-mannosidases, as well as binding proteins involved in sugar translocation (Fierer et al., 2007; Yu et al., 2020);. Therefore, soil *Proteobacteria*, *Actinobacteria*, *Firmicutes*, and *Bacteroidetes* dominated straw decomposition in our study. Crop straw is rich in cellulose, which is the most abundant renewable natural organic matter on earth, and *Pseudomonas* play a key role in cellulose decomposition (Kumar et al., 2015). The abundance of *Pseudomonas* in straw-amended treatments was significantly higher than that in the other treatments, which made the treatments accelerate straw decomposition.

Temperature accelerates straw decomposition by changing the composition of the soil microbial community, which in turn affects extracellular enzyme activities related to straw decomposition (Feng and Simpson, 2009; Yergeau et al., 2012; Stark et al., 2015). Wahdan et al. (2023) indicated that the main dominant phylum of soil bacterial community in the straw decomposition were *Proteobacteria*, *Actinobacteria*, and *Bacteroidetes*, and the abundance of *Enterobacteriaceae* and *Pseudomonas* showed an increasing trend with the increase of temperature. Previous studies on lignin-degrading microorganisms in tropical forest (high-temperature) soils have indicated that soil *Alphaproteobacteria* and *Gammaproteobacteria* are the main active flora (Olson et al., 2007; Jiang et al., 2015). In this study, we used DNA-SIP technology to investigate the changes in soil microbial communities during straw decomposition at different temperatures, which can accurately identify the microbial groups utilizing straw-C. Our results showed that the dominant phyla utilizing straw-C at different temperatures were *Proteobacteria* and *Actinobacteria* in the early stage of straw decomposition, and *Proteobacteria*, *Actinobacteria*, and *Firmicutes* in the later stage. Meanwhile, the *Gammaproteobacteria* (*Acidibacter* and *Lysobacter*) were the unique and predominant class involved in straw decomposition at medium and high temperatures (25°C and 35°C). Bao et al. (2021) found that *Actinobacteria* play important ecophysiological roles at various stages of straw decomposition and maintain functional composition during the decomposition process. Previous studies pointed out that *Gammaproteobacteria* is the relevant group of bacteria involved in cellulose degradation (assimilation of straw-C) and the main carbon-utilizing bacteria (Bernard et al., 2007; Eichorst and Kuske, 2012; Rime et al., 2016);. This shows that *Gammaproteobacteria* is one of the reasons for the fast rate of straw decomposition under medium- and high-temperature conditions. In addition, we found that the incubation temperature had a higher effect on soil microbial community composition than the fertilization treatments in our study (**Figures 2B, C**).

### 4.3 The important ecological roles of microorganisms utilizing straw as a carbon source

Plant residue decomposition processes and microbial communities are closely influenced by the functional characteristics of microorganisms (Wegner and Liesack, 2016; Guo et al., 2022; Zhang et al., 2022; Bao et al., 2023). Previous studies of DNA-SIP experiments have confirmed that soil bacteria contain large amounts of ^13^C labels not only as a result of cross-feeding in the experiments, but also due to their secretion of hydrolytic enzymes for actual degradation of plant residues (López-Mondéjar et al., 2018; Wilhelm et al., 2019; López-Mondéjar et al., 2020). Bao et al. (2023) found that the carbohydrate metabolism of bacteria decreased significantly with increasing soil fertility. López-Mondéjar et al. (2020) indicated that soil bacteria contain an abundance of genes encoding catabolic cellulases and hemicellulases, which play a key role in plant residue degradation, and that most of the CAZymes families have broad substrate specialization through DNA-SIP with shotgun metagenomic sequencing analysis. Our results partially support the above conclusion that the organic-amended treatments increased the relative abundance of GT and AA enzyme genes during the pre-straw decomposition period, and the higher contributions to CAZymes genes were from *Actinomycetes*, *Alphaproteobacteria*, and *Gammaproteobacteria*. Our result confirmed the second hypothesis. High-fertility soils were selected for fast-growing but inefficient bacteria, whereas low-fertility soils were selected for slow but efficient bacteria to degrade plant residues, and nutrient status influences the ecological strategies and metabolic trade-offs of bacteria due to the cytological economics of energy partitioning between growth rate and substrate utilization efficiency (Roller et al., 2016; Niederdorfer et al., 2017). *Actinomycetes* are highly competitive for carbon sources and adaptable to their environment, so they have the highest contribution to CAZymes genes (Swarnalakshmi et al., 2016; van Bergeijk et al., 2020). The presence of CBM enzyme genes in bacterial laccases permits direct binding of bacterial cells to the target polysaccharide, increasing the potency of the laccases and decreasing competitive rejection for cellulose degradation (Donohoe and Resch, 2015). In our study, the unique and predominant class of CAZymes at high temperature was the CBM, which may be one of the reasons for the fast rate of straw decomposition under high temperatures.

## 5 Conclusions

Before and after straw addition, organic-amended treatments, especially straw-amended treatments, increased soil bacterial *Alpha* diversity and the potential for resistance to changes in the external environment. The straw decomposition process was dominated by soil *Proteobacteria*, *Actinobacteria*, and *Firmicutes* under different incubation temperatures and fertilization treatments on the 7^th^ day and 30^th^ day of incubation. The effect of incubation temperature on soil microbial community composition was higher than that of fertilization treatments. Soil *Alphaproteobacteria* and *Actinomycetia* were responsible for dominating straw decomposition, and *Gammaproteobacteria* (*Pseudomonas*, *Steroidobacter*, *Acidibacter*, and *Arenimonas*) were responsible for accelerating straw decomposition. Compared to the chemical fertilizer application alone, organic-amended treatments, especially straw-amended treatments, increased the relative abundance of GT and AA enzyme genes and decreased the relative abundance of GH, CE, and PL enzyme genes. *Alphaproteobacteria*, *Actinomycetia*, and *Gammaproteobacteria* had higher relative contribution to carbohydrase genes. Fertilization treatments promote straw decomposition by increasing the abundance of indicator bacterial groups involved in straw decomposition, which is important for isolating key microbial species involved in straw decomposition under global warming.

## Data Availability

The datasets presented in this study can be found in online repositories. The names of the repository/repositories and accession number(s) can be found in the article/**Supplementary Material**.

## References

[B1] Ai C. Zhang S. Zhang X. Guo D. Zhou W. Huang S. (2018). Distinct responses of soil bacterial and fungal communities to changes in fertilization regime and crop rotation. Geoderma 319, 156–166. doi: 10.1016/j.geoderma.2018.01.010

[B2] Banerjee S. Schlaeppi K. van der Heijden M. G. (2018). Keystone taxa as drivers of microbiome structure and functioning. Nat. Rev. Microbiol. 16, 567–576. doi: 10.1038/s41579-018-0024-1 29789680

[B3] Bao Y. Dolfing J. Chen R. Li Z. Lin X. Feng Y. (2023). Trade-off between microbial ecophysiological features regulated by soil fertility governs plant residue decomposition. Soil Tillage Res. 229, 105679. doi: 10.1016/j.still.2023.105679

[B4] Bao Y. Dolfing J. Guo Z. Chen R. Wu M. Li Z.. (2021). Important ecophysiological roles of non-dominant Actinobacteria in plant residue decomposition, especially in less fertile soils. Microbiome 9, 1–17. doi: 10.1186/s40168-021-01032-x 33827695 PMC8028251

[B5] Bei S. Zhang Y. Li T. Christie P. Li X. Zhang J. (2018). Response of the soil microbial community to different fertilizer inputs in a wheat-maize rotation on a calcareous soil. Agriculture Ecosyst. Environ. 260, 58–69. doi: 10.1016/j.agee.2018.03.014

[B6] Bernard L. Mougel C. Maron P. A. Nowak V. Lévêque J. Henault C.. (2007). Dynamics and identification of soil microbial populations actively assimilating carbon from 13C-labelled wheat residue as estimated by DNA-and RNA-SIP techniques. Environ. Microbiol. 9, 752–764. doi: 10.1111/j.1462-2920.2006.01197.x 17298374

[B7] Blesh J. Ying T. (2020). Soil fertility status controls the decomposition of litter mixture residues. Ecosphere 11, e03237. doi: 10.1002/ecs2.v11.8

[B8] Bradford M. A. Veen G. F. Bonis A. Bradford E. M. Classen A. T. Cornelissen J. H. C.. (2017). A test of the hierarchical model of litter decomposition. Nat. Ecol. Evol. 1, 1836–1845. doi: 10.1038/s41559-017-0367-4 29133902

[B9] Buchfink B. Xie C. Huson D. H. (2015). Fast and sensitive protein alignment using DIAMOND. Nat. Methods 12, 59–60. doi: 10.1038/nmeth.3176 25402007

[B10] Chen Y. Murrell J. C. (2010). When metagenomics meets stable-isotope probing: progress and perspectives. Trends Microbiol. 18, 157–163. doi: 10.1016/j.tim.2010.02.002 20202846

[B11] Cornwell W. K. Cornelissen J. H. Amatangelo K. Dorrepaal E. Eviner V. T. Godoy O.. (2008). Plant species traits are the predominant control on litter decomposition rates within biomes worldwide. Ecol. Lett. 11, 1065–1071. doi: 10.1111/j.1461-0248.2008.01219.x 18627410

[B12] Delgado-Baquerizo M. Giaramida L. Reich P. B. Khachane A. N. Hamonts K. Edwards C.. (2016). Lack of functional redundancy in the relationship between microbial diversity and ecosystem functioning. J. Ecol. 104, 936–946. doi: 10.1111/jec.2016.104.issue-4

[B13] Donohoe B. S. Resch M. G. (2015). Mechanisms employed by cellulase systems to gain access through the complex architecture of lignocellulosic substrates. Curr. Opin. Chem. Biol. 29, 100–107. doi: 10.1016/j.cbpa.2015.08.014 26529490

[B14] Edgar R. C. (2013). UPARSE: highly accurate OTU sequences from microbial amplicon reads. Nat. Methods 10, 996–998. doi: 10.1038/nmeth.2604 23955772

[B15] Eichorst S. A. Kuske C. R. (2012). Identification of cellulose-responsive bacterial and fungal communities in geographically and edaphically different soils by using stable isotope probing. Appl. Environ. Microbiol. 78, 2316–2327. doi: 10.1128/AEM.07313-11 22287013 PMC3302591

[B16] Fan F. Yu B. Wang B. George T. S. Yin H. Li D.. (2019). Microbial mechanisms of the contrast residue decomposition and priming effect in soils with different organic and chemical fertilization histories. Soil Biol. Biochem. 135, 213–221. doi: 10.1016/j.soilbio.2019.05.001

[B17] Fang Y. Nazaries L. Singh B. K. Singh B. P. (2018). Microbial mechanisms of carbon priming effects revealed during the interaction of crop residue and nutrient inputs in contrasting soils. Global Change Biol. 24, 2775–2790. doi: 10.1111/gcb.2018.24.issue-7 29603502

[B18] Fazi S. Amalfitano S. Pernthaler J. Puddu A. (2005). Bacterial communities associated with benthic organic matter in headwater stream microhabitats. Environ. Microbiol. 7, 1633–1640. doi: 10.1111/j.1462-2920.2005.00857.x 16156736

[B19] Feng X. Simpson M. J. (2009). Temperature and substrate controls on microbial phospholipid fatty acid composition during incubation of grassland soils contrasting in organic matter quality. Soil Biol. Biochem. 41, 804–812. doi: 10.1016/j.soilbio.2009.01.020

[B20] Fierer N. Bradford M. A. Jackson R. B. (2007). Toward an ecological classification of soil bacteria. Ecology 88, 1354–1364. doi: 10.1890/05-1839 17601128

[B21] Glassman S. I. Weihe C. Li J. Albright M. B. Looby C. I. Martiny A. C.. (2018). Decomposition responses to climate depend on microbial community composition. Proc. Natl. Acad. Sci. 115, 11994–11999. doi: 10.1073/pnas.1811269115 30397146 PMC6255157

[B22] Gonthier D. J. Ennis K. K. Farinas S. Hsieh H. Y. Iverson A. L. Batáry P.. (2014). Biodiversity conservation in agriculture requires a multi-scale approach. Proc. R. Soc. B: Biol. Sci. 281, 20141358. doi: 10.1098/rspb.2014.1358 PMC4132690 25100703

[B23] Guo T. Zhang Q. Song D. Ai C. Zhang S. Yue K.. (2022). Varying microbial utilization of straw-derived carbon with different long-term fertilization regimes explored by DNA stable-isotope probing. Eur. J. Soil Biol. 108, 103379. doi: 10.1016/j.ejsobi.2021.103379

[B24] Helmke P. A. Sparks D. L. (1996). “Lithium, sodium, potassium, rubidium and cesium,” in Methods of Soil Analysis Part 3: Chemical Methods. Eds. Sparks D. L. Page A. L. Helmke P. A. Loeppert R. H. Soltanpour P. N. Tabatabai M. A. Johnston C. T. Sumner M. E. (SSSA, Madison, WI, USA), 551–574.

[B25] Huang S. W. (2019). Principle and Technology of Green and High Effciency Precision Fertilization for Greenhouse Vegetables (Beijing, China: China Agricultural Science and Technology Press).

[B26] Jiang L. Song M. Luo C. Zhang D. Zhang G. (2015). Novel phenanthrene-degrading bacteria identified by DNA-stable isotope probing. PloS One 10, e0130846. doi: 10.1371/journal.pone.0130846 26098417 PMC4476716

[B27] Jin N. Lyu J. Yu J. (2022). Reduced chemical fertilizer combined with bio-organic fertilizer affects the soil microbial community and yield and quality of lettuce. Front. Microbiol. 13, 863325. doi: 10.3389/fmicb.2022.863325 35531292 PMC9069001

[B28] Kamble P. N. Gaikwad V. B. Kuchekar S. R. Bååth E. (2014). Microbial growth, biomass, community structure and nutrient limitation in high pH and salinity soils from Pravaranagar (India). Eur. J. Soil Biol. 65, 87–95. doi: 10.1016/j.ejsobi.2014.10.005

[B29] Kong Y. Kuzyakov Y. Ruan Y. Zhang J. Wang T. Wang M.. (2020). DNA stable-isotope probing delineates carbon flows from rice residues into soil microbial communities depending on fertilization. Appl. Environ. Microbiol. 86, e02151–e02119. doi: 10.1128/AEM.02151-19 31953339 PMC7082572

[B30] Kumar M. Revathi K. Khanna S. (2015). Biodegradation of cellulosic and lignocellulosic waste by Pseudoxanthomonas sp R-28. Carbohydr. polymers 134, 761–766. doi: 10.1016/j.carbpol.2015.08.072 26428183

[B31] Li S. Lü S. Zhang Y. Liu Y. Gao Y. Ao Y. (2015). The change of global terrestrial ecosystem net primary productivity (NPP) and its response to climate change in CMIP5. Theor. Appl. Climatology 121, 319–335. doi: 10.1007/s00704-014-1242-8

[B32] López-Mondéjar R. Brabcova V. Stursova M. Davidova A. Jansa J. Cajthaml T.. (2018). Decomposer food web in a deciduous forest shows high share of generalist microorganisms and importance of microbial biomass recycling. ISME J. 12, 1768–1778. doi: 10.1038/s41396-018-0084-2 29491492 PMC6018761

[B33] López-Mondéjar R. Tláskal V. Větrovský T. Štursová M. Toscan R. da Rocha U. N.. (2020). Metagenomics and stable isotope probing reveal the complementary contribution of fungal and bacterial communities in the recycling of dead biomass in forest soil. Soil Biol. Biochem. 148, 107875. doi: 10.1016/j.soilbio.2020.107875

[B34] Luan H. Gao W. Huang S. Tang J. Li M. Zhang H.. (2020). Substitution of manure for chemical fertilizer affects soil microbial community diversity, structure and function in greenhouse vegetable production systems. PloS One 15, e0214041. doi: 10.1371/journal.pone.0214041 32084129 PMC7034837

[B35] Lupatini M. Korthals G. W. Janssens T. K. Kuramae E. E. (2017). Soil microbiome is more heterogeneous in organic than in conventional farming system. Front. Microbiol. 7, 237546. doi: 10.3389/fmicb.2016.02064 PMC5209367 28101080

[B36] Ma L. Li R. Luan H. Tang J. Wang L. Huang S. (2024). Temperature matters more than fertilization for straw decomposition in the soil of greenhouse vegetable field. Agronomy 14, 233. doi: 10.3390/agronomy14020233

[B37] Marschner P. Umar S. Baumann K. (2011). The microbial community composition changes rapidly in the early stages of decomposition of wheat residue. Soil Biol. Biochem. 43, 445–451. doi: 10.1016/j.soilbio.2010.11.015

[B38] Mi W. Wu L. Brookes P. C. Liu Y. Zhang X. Yang X. (2016). Changes in soil organic carbon fractions under integrated management systems in a low-productivity paddy soil given different organic amendments and chemical fertilizers. Soil Tillage Res. 163, 64–70. doi: 10.1016/j.still.2016.05.009

[B39] Murase J. Shibata M. Lee C. G. Watanabe T. Asakawa S. Kimura M. (2012). Incorporation of plant residue–derived carbon into the microeukaryotic community in a rice field soil revealed by DNA stable-isotope probing. FEMS Microbiol. Ecol. 79, 371–379. doi: 10.1111/j.1574-6941.2011.01224.x 22092599

[B40] Niederdorfer R. Besemer K. Battin T. J. Peter H. (2017). Ecological strategies and metabolic trade-offs of complex environmental biofilms. NPJ Biofilms Microbiomes 3, 21. doi: 10.1038/s41522-017-0029-y 28955480 PMC5612939

[B41] Norman R. J. Edberg J. C. Stucki J. W. (1985). Determination of nitrate in soil extracts by dual-wavelength ultraviolet spectrophotometry. Soil Sci. Soc. America J. 49, 1182–1185. doi: 10.2136/sssaj1985.03615995004900050022x

[B42] Olsen S. R. Cole C. V. Watanabe F. S. Dean L. A. (1954). Estimation of available phosphorus in soils by extraction with sodium bicarbonate. USDA Circ. 939, 1–19.

[B43] Olson P. E. Castro A. Joern M. DuTeau N. M. Pilon-Smits E. A. Reardon K. F. (2007). Comparison of plant families in a greenhouse phytoremediation study on an aged polycyclic aromatic hydrocarbon–contaminated soil. J. Environ. Qual. 36, 1461–1469. doi: 10.2134/jeq2006.0371 17766825

[B44] Quast C. Pruesse E. Yilmaz P. Gerken J. Schweer T. Yarza P.. (2012). The SILVA ribosomal RNA gene database project: improved data processing and web-based tools. Nucleic Acids Res. 41, D590–D596. doi: 10.1093/nar/gks1219 23193283 PMC3531112

[B45] Reilly K. Cullen E. Lola-Luz T. Stone D. Valverde J. Gaffney M.. (2013). Effect of organic, conventional and mixed cultivation practices on soil microbial community structure and nematode abundance in a cultivated onion crop. J. Sci. Food Agric. 93, 3700–3709. doi: 10.1002/jsfa.2013.93.issue-15 23633428

[B46] Rime T. Hartmann M. Stierli B. Anesio A. M. Frey B. (2016). Assimilation of microbial and plant carbon by active prokaryotic and fungal populations in glacial forefields. Soil Biol. Biochem. 98, 30–41. doi: 10.1016/j.soilbio.2016.03.012

[B47] Roller B. R. Stoddard S. F. Schmidt T. M. (2016). Exploiting rRNA operon copy number to investigate bacterial reproductive strategies. Nat. Microbiol. 1, 1–7. doi: 10.1038/nmicrobiol.2016.160 PMC5061577 27617693

[B48] Rong Q. L. Li R. N. Huang S. W. Tang J. W. Zhang Y. C. Wang L. Y. (2018). Soil microbial characteristics and yield response to partial substitution of chemical fertilizer with organic amendments in greenhouse vegetable production. J. Integr. Agric. 17, 1432–1444. doi: 10.1016/S2095-3119(18)61946-X

[B49] Sanaullah M. Chabbi A. Maron P. A. Baumann K. Tardy V. Blagodatskaya E.. (2016). How do microbial communities in top-and subsoil respond to root litter addition under field conditions? Soil Biol. Biochem. 103, 28–38. doi: 10.1016/j.soilbio.2016.07.017

[B50] Stark S. Männistö M. K. Ganzert L. Tiirola M. Häggblom M. M. (2015). Grazing intensity in subarctic tundra affects the temperature adaptation of soil microbial communities. Soil Biol. Biochem. 84, 147–157. doi: 10.1016/j.soilbio.2015.02.023

[B51] Swarnalakshmi K. Senthilkumar M. Ramakrishnan B. (2016). “Endophytic Actinobacteria: nitrogen fixation, phytohormone production, and antibiosis,” in Plant growth promoting Actinobacteria: a vew avenue for enhancing the productivity and soil fertility of grain legumes. Eds. Subramaniam G. Arumugam S. Rajendran V. (Springer Singapore, Singapore), 123–145.

[B52] Tardy V. Chabbi A. Charrier X. de Berranger C. Reignier T. Dequiendt S.. (2015). Land use history shifts in *situ* fungal and bacterial successions following wheat straw inut into soil. PloS One 10, e0130672. doi: 10.1371/journal.pone.0130672 26102585 PMC4478037

[B53] Tveit A. Schwacke R. Svenning M. M. Urich T. (2013). Organic carbon transformations in high-Arctic peat soils: key functions and microorganisms. ISME J. 7, 299–311. doi: 10.1038/ismej.2012.99 22955232 PMC3554415

[B54] van Bergeijk D. A. Terlouw B. R. Medema M. H. van Wezel G. P. (2020). Ecology and genomics of Actinobacteria: new concepts for natural product discovery. Nat. Rev. Microbiol. 18, 546–558. doi: 10.1038/s41579-020-0379-y 32483324

[B55] Wahdan S. F. M. Ji L. Schädler M. Wu Y. T. Sansupa C. Tanunchai B.. (2023). Future climate conditions accelerate wheat straw decomposition alongside altered microbial community composition, assembly patterns, and interaction networks. ISME J. 17, 238–251. doi: 10.1038/s41396-022-01336-2 36352255 PMC9860053

[B56] Walker T. W. N. Kaiser C. Strasser F. Herbold C. W. Leblans N. I. W. Woebken D.. (2018). Microbial temperature sensitivity and biomass change explain soil carbon loss with warming. Nat. Climate Change 8, 885–889. doi: 10.1038/s41558-018-0259-x PMC6166784 30288176

[B57] Wang M. Ni H. Liu Y. Zhang Y. Jiang L. Tu Q. (2023). Effect of fertilization combination on cucumber quality and soil microbial community. Front. Microbiol. 14, 1122278. doi: 10.3389/fmicb.2023.1122278 36910239 PMC9996052

[B58] Wardle D. A. Bardgett R. D. Klironomos J. N. Setala H. van der Putten W. H. Wall D. H. (2004). Ecological linkages between aboveground and belowground biota. Science 304, 1629–1633. doi: 10.1126/science.1094875 15192218

[B59] Wegner C. E. Liesack W. (2016). Microbial community dynamics during the early stages of plant polymer breakdown in paddy soil. Environ. Microbiol. 18, 2825–2842. doi: 10.1111/emi.2016.18.issue-9 25712035

[B60] Wei M. Hu G. Wang H. Bai E. Lou Y. Zhang A.. (2017). 35 years of manure and chemical fertilizer application alters soil microbial community composition in a Fluvo-aquic soil in Northern China. Eur. J. Soil Biol. 82, 27–34. doi: 10.1016/j.ejsobi.2017.08.002

[B61] Wilhelm R. C. Singh R. Eltis L. D. Mohn W. W. (2019). Bacterial contributions to delignification and lignocellulose degradation in forest soils with metagenomic and quantitative stable isotope probing. ISME J. 13, 413–429. doi: 10.1038/s41396-018-0279-6 30258172 PMC6331573

[B62] Xu Y. Ding F. Gao X. Wang Y. Li M. Wang J. (2019). Mineralization of plant residues and native soil carbon as affected by soil fertility and residue type. J. Soils Sediments 19, 1407–1415. doi: 10.1007/s11368-018-2152-7

[B63] Yang X. Wang Y. Xu Q. Liu W. Liu L. Wu Y.. (2021). Soil fertility underlies the positive relationship between island area and litter decomposition in a fragmented subtropical forest landscape. Catena 204, 105414. doi: 10.1016/j.catena.2021.105414

[B64] Ye G. Fan J. Hu H. W. Chen J. Zhong X. Chen J.. (2022). Short-term cellulose addition decreases microbial diversity and network complexity in an Ultisol following 32-year fertilization. Agriculture Ecosyst. Environ. 325, 107744. doi: 10.1016/j.agee.2021.107744

[B65] Yergeau E. Bokhorst S. Kang S. Zhou J. Greer C. W. Aerts R.. (2012). Shifts in soil microorganisms in response to warming are consistent across a range of Antarctic environments. ISME J. 6, 692–702. doi: 10.1038/ismej.2011.124 21938020 PMC3282189

[B66] Yu J. Pavia M. J. Deem L. M. Crow S. E. Penton C. R. (2020). DNA-stable isotope probing shotgun metagenomics reveals the resilience of active microbial communities to biochar amendment in oxisol soil. Front. Microbiol. 11, 587972. doi: 10.3389/fmicb.2020.587972 33329461 PMC7717982

[B67] Zhan Y. Liu W. Bao Y. Zhang J. Petropoulos E. Li Z.. (2018). Fertilization shapes a well-organized community of bacterial decomposers for accelerated paddy straw degradation. Sci. Rep. 8, 7981. doi: 10.1038/s41598-018-26375-8 29789525 PMC5964224

[B68] Zhang Q. Guo T. Sheng K. Shi W. Han Y. Wang Y.. (2022). Continuous straw return for 8 years mitigates the negative effects of inorganic fertilisers on C-cycling soil bacteria. Eur. J. Soil Sci. 73, e13322. doi: 10.1111/ejss.13322

[B69] Zhang W. L. Kolbe H. Zhang R. L. (2020). Research progress of SOC functions and transformation mechanisms. Scientia Agricultura Sin. 53, 371–331. doi: 10.3864/j.issn.0578-1752.2020.02.007

[B70] Zhao S. Qiu S. Xu X. Ciampitti I. A. Zhang S. He P. (2019). Change in straw decomposition rate and soil microbial community composition after straw addition in different long-term fertilization soils. Appl. Soil Ecol. 138, 123–133. doi: 10.1016/j.apsoil.2019.02.018

[B71] Zheng T. Xie H. Thompson G. L. Bao X. Deng F. Yan E.. (2021). Shifts in microbial metabolic pathway for soil carbon accumulation along subtropical forest succession. Soil Biol. Biochem. 160, 108335. doi: 10.1016/j.soilbio.2021.108335

[B72] Zhou G. Zhang J. Zhang C. Feng Y. Chen L. Yu Z.. (2016). Effects of changes in straw chemical properties and alkaline soils on bacterial communities engaged in straw decomposition at different temperatures. Sci. Rep. 6, 22186. doi: 10.1038/srep22186 26916902 PMC4768159

