# Supplementary material for: Impacts of long-term different fertilization regimes on microbial utilization of straw-derived carbon in greenhouse vegetable soils: insights from its ecophysiological roles and temperature responses
Source: Front Plant Sci. 2024 Oct 25;15:1486817. doi: 10.3389/fpls.2024.1486817 (PMC11543410; doi:10.3389/fpls.2024.1486817)
Supplement: Supplementary file 1 [file Table1.docx]

Table S1 Nitrogen and carbon inputs in each fertilization treatment during the winter-spring cucumber season and autumn-winter tomato season (kg hm^-2^)

| Treatments | Nitrogen input | | | | Carbon input | | | |
| --- | --- | --- | --- | --- | --- | --- | --- | --- |
|  | Chemical fertilizer | Organic manure | Corn straw | Total | Organic manure | Corn straw | | Total |
| winter-spring cucumber season | | | | | | | | |
| CF | 600.0 | 0 | 0 | 600.0 | 0 | 0 | 0 | |
| CM | 300.0 | 300.0 | 0 | 600.0 | 2566.4 | 0 | 2566.4 | |
| CMS | 300.0 | 150.0 | 150.0 | 600.0 | 1283.2 | 6482.8 | 7766.0 | |
| CS | 300.0 | 0 | 300.0 | 600.0 | 0 | 12965.5 | 12965.5 | |
| autumn-winter tomato season | | | | | | | | |
| CF | 450.0 | 0 | 0 | 450.0 | 0 | 0 | 0 | |
| CM | 225.0 | 225.0 | 0 | 450.0 | 1924.8 | 0 | 1924.8 | |
| CMS | 225.0 | 112.5 | 112.5 | 450.0 | 962.4 | 4862.1 | 5824.5 | |
| CS | 225.0 | 0 | 225.0 | 450.0 | 0 | 9724.2 | 9724.2 | |
